# Prevalence, incidence and seroconversion of HIV and Syphilis infections among pregnant women of South Africa

**DOI:** 10.4102/sajid.v36i1.296

**Published:** 2021-11-24

**Authors:** Monjurul Hoque, Muhammad E. Hoque, Guido van Hal, Somaya Buckus

**Affiliations:** 1 Kwadabeka Community Health Centre, Durban, South Africa; 2 Research Department, Management College of Southern Africa, Durban, South Africa; 3 Department of Social Epidemiology and Health Policy, University of Antwerp, Antwerp, Belgium

**Keywords:** antenatal care, booking visit, Kwadabeka, rapid test, HIV, syphilis

## Abstract

**Background:**

Pregnant women in South Africa suffer from HIV and syphilis infections resulting in negative pregnancy outcomes. Little is known about the prevalence, incidence, seroconversion, and associated risk factors for those attending a midwife run obstetric unit.

**Methods:**

A retrospective cohort study was undertaken among pregnant women attending antenatal clinic from January to December 2018. Logistic regression was conducted to determine the risk factors for HIV and syphilis.

**Results:**

The prevalence of HIV and syphilis were 44.3% (95% confidence interval [CI]; 41.6:46.7) and 3.8% (95% CI; 3.1:4.1), respectively. The seroconversion and incidence for HIV were 4.0% (95% CI; 3.6:4.6) and 17.1 per 100 person-years, and for syphilis 2.6% (95% CI; 2.3:2.8) and 10.9 per 100 person-years, respectively. Significant predictors for HIV prevalence were ages: ages < 20 years, Odds ratio (OR) = 0.11 (*p* < 0.05), ages 20–24 years, OR = 0.19 (*p* < 0.05) and ages 25–29 years, OR = 0.38 (*p* < 0.05); gestational age: second trimester, OR = 0.68 (*p* < 0.05) and non-reactive syphilis, OR = 0.45 (*p* < 0.05). Age was the predictor for HIV incidence or seroconversion (age < 20 year, OR = 0.12, *p* = 0.01). Predictors for syphilis were ages < 20 years, OR = 0.11 (*p* < 0.05), ages 20–24 years and HIV status. Gestational age > 27 weeks were nine times (OR = 9.2, *p* = 0.03) more likely to seroconvert to syphilis.

**Conclusions:**

The present study found high rates of seroprevalence, seroconversion and incidence of HIV and syphilis among pregnant women.

## Introduction

Human Immunodeficiency Virus (HIV) and syphilis infections are continuing as a public health concern in developing countries, especially in South Africa (SA). In SA, there are over 7.5 million people living with HIV, causing the highest disease burden in the world, of which 4.7 million women are aged 19 years and above.^1^ The incidence of HIV and syphilis are known to be higher among pregnant women than in non-pregnant women and both infections do coexist.^2,3,4^ Both of these infections are known to cause adverse pregnancy outcomes such as stillbirth, low birth weight of babies and maternal deaths, if left untreated.^5^ Pregnant women are sexually active and known to have a higher risk of acquiring both the HIV and syphilis infections from their infected partners.^6^ It is praiseworthy to note that SA has the highest anti-retroviral treatment (ART) programme that 5.2 million people have received by 2019.^7^

More than 95% pregnant women in SA attend the public health facilities for antenatal care (ANC) and receive ART, which also works as a preventive measure for mother-to-child transmission of HIV.^1^ The effective diagnosis and simple treatment for syphilis are available at present. *Treponema pallidum*, a bacterium is the causative organism for syphilis. In 2019, approximately 200 000 stillbirths and neonatal deaths were reported by the World Health Organization (WHO) because of congenital syphilis and thus ranked as the second leading cause of preventable stillbirth worldwide.^7^ It is a routine practice in SA that screening for HIV and syphilis are undertaken at the first and subsequent ANC visits according to the SA national policy for appropriate intervention.^8^ World Health Organization has promoted a strategic plan to combat and eliminate congenital syphilis around the globe by recommending prevention of transmission from mother to child through routine screening and treating of syphilis to reduce the incidence by 90%, and to keep control of congenital syphilis < 50 cases per 100 000 live births.^9,10^ Therefore, the strengthening of ANC became fundamental in screening and treating syphilis promptly and appropriately during ANC.^11^ The WHO strategies in eliminating congenital syphilis rest on four principles.^7^ Among them, the most important health service-related ones are to increase access to quality maternal health services, screen and treat both pregnant women and their partners and finally, to ensure monitoring and evaluation at different spheres of governance.^7^

To monitor the temporal trends of HIV epidemic and syphilis, the National Department of Health SA (DOH) undertakes National Antenatal Sentinel HIV and Syphilis seroprevalence Surveys among pregnant women. The last survey conducted in 2017 highlights different rates of HIV prevalence (national 30.7%) and a great variation between SA provinces exist. The KwaZulu-Natal province (KZN) has the highest rate of 41.1%.^12^ The lowest prevalence of syphilis was 0.4% in KZN and 1.6% nationally in SA during 2011.^13^ The National Institute for Communicable Disease (NICD) of SA reported higher rates of syphilis among pregnant women attending ANC ranging from 1.1% to 4.6% with approximately 72% of them being screened for syphilis.^12,14^ It is noted that significant variation of HIV and syphilis prevalence are found between provinces of SA. Monitoring the incidence and prevalence of HIV and syphilis among pregnant women and identifying risk factors of pregnant women can facilitate evaluation of existing intervention programmes. There is little knowledge regarding the prevalence of syphilis and HIV seroconversion, and the risk factors among pregnant women attending a Midwife run Obstetric Unit (MOU) in KZN. It is thus aimed to provide information to help managers to plan for effective strategies to treat and control HIV and syphilis infections during pregnancy. The objectives are to estimate the prevalence, incidence, seroconversion rates, and risk factors for HIV and syphilis during the antenatal period among pregnant women.

## Method and materials

### Study design

A retrospective cohort study was undertaken among the antenatal clinic attendees using the clinic register. All pregnant women who attended for booking visits between January and December 2018 were included and followed up until their last visits to the clinic.

### Setting and population

This study was undertaken at a Primary Health Care (PHC) set up of Kwadabeka Community Health Centre (KCHC). It provides first level care to over 150 000 predominantly Zulu-speaking black African populations living in the outskirts of Durban, which is known to be a peri-urban community. The residences are reliant on KCHC as a public health facility that provides free services to its clients. Since 2015, antiretroviral treatment (ART) has been made available at KCHC for all HIV-positive pregnant women.^15^ Unpublished data from the clinic annual report shows that the total and maternity related headcounts are 240 000 and 5000 respectively. The facility also conducts about 700 deliveries annually. Trained and skilled midwives provide maternity services 24 h a day. However, the routine ANC is provided as a day service between 07h00 and 16h00. Both ANC and delivery services are rendered using the DOH protocol and guidelines for midwives that was developed in 2002 and reviewed in 2015.^16^

### Data sources, screening and management of HIV and Syphilis infections

Data were collected from the antenatal clinic register (ACR). The manual ACR was developed and implemented by the national department of health that catered for the recording of selective ANC findings of all (booking and follow up) visits in a structured format.^16^ The detailed ANC findings are recorded in the maternity case record. The maternity case record is given back to the pregnant woman for future attendance of ANC and delivery services. At the first (booking) and subsequent antenatal visits, the midwives recorded the results of syphilis and HIV screening test results together with other demographic, obstetric and other general medical histories, and examination findings in the ACR.^16^ The ACR remains with the clinic for recording and reporting purposes. The ACR contains among others the age in years, parity, gravidity, previous obstetric and medical histories and gestational age (GA) of the women. The GA was measured from symphysis pubis to the fundus of the uterus in centimetres or using the last menstrual period. The researchers obtained the ACR from the KCHC antenatal clinic and captured manual data to electronic format (Microsoft Excel) with double entry to minimise errors by the research assistant who was trained in using the Microsoft Excel programme.

All pregnant women were counselled and given information about antenatal serological screening tests at their first and follow-up visits. The routine practice at KCHC is that all mothers are screened for anaemia, HIV, syphilis, and rhesus factors and recorded the results in the ACR. The syphilis screening test at the booking and the rescreening in the third trimester or later visits were recorded as ‘reactive or non-reactive’. In accordance with the DOH policy on HIV screening and treating purposes, finger prick blood samples were tested with two rapid tests of two different suppliers. If the first test became positive, a second rapid test using a different test kit from a different supplier was performed. If the first test became positive, a second rapid test using a different test kit from a different supplier was performed. If both the tests became negative, the mother was considered HIV negative. If both the test results were positive, the mother was considered HIV positive. If one test was negative, then a confirmatory Enzyme-Linked Immunosorbent Assay (ELISA) test was performed at the laboratory. Every confirmed positive HIV woman was recorded as HIV status positive and offered ART using highly active ART (HAART) on the same day as part of the universal ART programme in SA should the client accepted ART. Those pregnant women who had HIV negative results were rescreened after 12 weeks or later when they became available for a repeat ANC visit. The Rapid Plasma Reagin (RPR) test was used to yield reactive or non-reactive results for syphilis. For the consideration of syphilis, the occurrence of one of these situations was considered: a record of a reactive result in the ACR and or the mother was treated with penicillin, as it was ‘syphilis positive or exposed’ (case definition). Those women who had non-reactive RPR results were re-screened at or after 32 weeks of GA, and the results recorded accordingly.

### Data analysis

Data were captured in Microsoft Excel (for Windows) and exported to Statistical Package for the Social Sciences (SPSS) (version 22.0) for analysis. Ages of the pregnant women were categorised into; < 20 years (teenage), 20–24 years, 25–29 years, 30–34 years and ≥ 35 years, GA into first trimester (0–13 weeks), second trimester (14–27 weeks) and third trimester (≥ 28 weeks). Parity was categorised as nil-parity (primipara or primigravida), parity 1–2, 3–4 and ≥ 5 (grandmultiparity). The screening results of HIV and syphilis at the booking and subsequent visits were used to estimate the prevalence of HIV and syphilis. The 12 weeks or later follow-up visit retest data were used for the estimation of seroconversion and incidence of HIV. For syphilis, the rescreening results at or around 32 weeks GA follow-up visits were used for estimation of seroconversion and incidence of maternal syphilis. We had calculated total person years followed up for repeat HIV and syphilis tests and measured incidence of HIV and syphilis per 100 person-years. Categorical variables were presented using frequencies and proportions. Differences in proportions of syphilis and HIV for different demographic and obstetric variables at the booking visit were examined using Pearson chi-square (χ^2^) and *p*-values. Step by step (backward) binary logistic regression analysis was conducted to determine the predictors for syphilis and HIV prevalence and incidence. Regression results were presented with adjusted odds ratio (OR) with corresponding 95% confidence intervals (95% CI) and *p*-values. *p*-values less than 0.05 were considered statistically significant.

### Ethical considerations

Umgungundlovu Health Ethics Review Board approved the study protocol (Reference no. UHERB 015/2020). Written permission from the Management of the KCHC was obtained to use the AR for the study. Informed consent was waived as we used secondary data. Full confidentiality and privacy were maintained to present the report.

## Results

### Demographic and pregnancy related information

**FIGURE 1:**
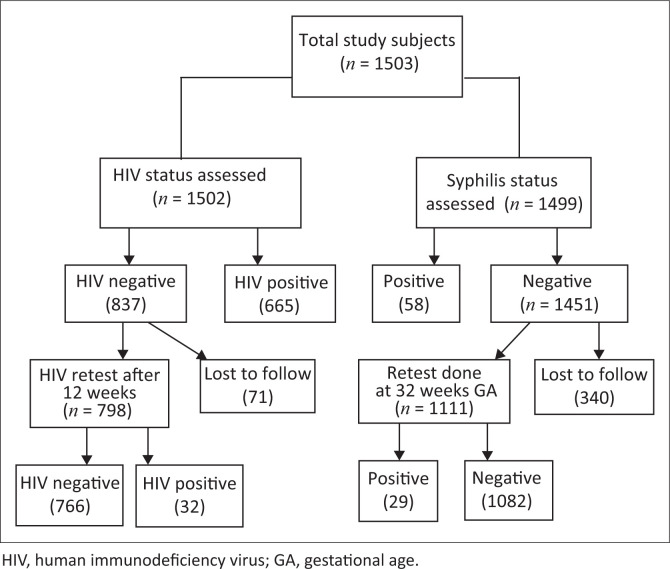
Flow diagram of study subjects at different stages of analysis.

A total of 1503 pregnant women had booking visits at KCHC for the study period. The information on ANC booking visit and follow-up at ANC clinic of pregnant women are shown in Figure 1. The median age and GA with interquartile ranges (IQR) were 25 (21–29) years and 22 (18–27) weeks, respectively. The majority of the participants belonged to the 20–29 years age group (56.3%), second trimester (62.7%) of GA (13–27 weeks) and had parity of 1–2 (52.6%) (Table 1).

**TABLE 1:** Demographic, obstetric and outcome variables of the study sample at booking visit.

Variables	Frequency	%	95% CI
**Age category (*n* = 1491) (years)**			
< 20	271	18.2	-
20–24	466	31.3	-
25–29	391	26.2	-
30–34	248	16.6	-
≥ 35	115	7.7	-
**Gestational age (*n* = 1365) (weeks)**			
Up to 13	190	13.9	-
14–27	854	62.6	-
≥ 28	321	23.5	-
**Parity (*n* = 1495)**			
Nil	461	30.8	-
1–2	831	55.6	-
3–4	186	12.4	-
≥ 5	17	1.1	-
**HIV status (*n* = 1502)**			
Negative	837	55.7	-
Positive	665	44.3	41.6–46.7
**HIV Positive and on ART (*n* = 665)**	662	99.5	-
**Seroconversion of HIV after 12 weeks (*n*= 798)**			
Yes	32	4.0	3.6– 4.6
HIV incidence (per 100 person-year)	32	17.3	11.2–22.1
**RPR status at booking visit (*n* = 1499)**			
Negative	1441	96.8	-
Positive	58	3.8	3.1–4.1
**RPR results at 32 weeks (*n* = 1111)**			
Positive (Seroconversion)	29	2.6	2.3–2.8
Syphilis incidence (per 100 person-year)	29	10.9	7.1–14.6

HIV, human immunodeficiency virus; RPR, Rapid Plasma Reagin; ART, anti-retroviral treatment; CI, confidence interval.

### Human immunodeficiency virus seroprevalence, seroconversion, incidence, and risk factors

Table 1 shows the prevalence of HIV at the booking visit of 44.3% (95% CI: 41.6% – 46.7%). Of those infected with HIV, virtually all (99.5%) accepted ART. Of those pregnant women (837) found to be HIV negative at the booking visit, 95.3% (798) were retested after 12 weeks. Thirty-two of them (798) became HIV positive, resulted seroconversion rate of 4.0% (95% CI; 3.6%:4.6%). Those 798 women were followed-up for 12 weeks and resulted in 184 person-years with 32 new occurrences of HIV resulted the incidence of 17.3 (95% CI; 11.1:22.3) per 100 person-years. Table 2 shows that HIV prevalence was significantly higher (75.7%) among older age group (≥ 35 years) followed by 64.5% and 51.7% among 30–34 years and 25–29 years age groups, respectively (*p* < 0.05). Gestational age and parity were significantly associated with HIV status at the booking visit (*p* < 0.05). Results showed that higher rates of HIV (49% and 61%) were found among those women in their third trimester and women with multiparity, respectively. The HIV status was also significantly associated with syphilis status (*p* < 0.05).

**TABLE 2:** Cross table analysis of syphilis and human immunodeficiency virus prevalence with demographic and obstetric variables at the booking visit with Chi-square and *p*-values.

Variables	Syphilis positive		*p*	HIV status: Positive		*p*
	*n/N*	%		*n*	%	
**Age category (*n* = 1491) (years)**	-	-	-	-	-	0.001
< 20	4	1.5	0.001	57	21.0	-
20–24	13	2.8	-	155	33.3	-
25–29	12	3.1	-	201	51.7	-
30–34	13	5.2	-	160	64.5	-
≥ 35	14	12.2	-	87	75.7	-
**Gestational age (*n* = 1365) (weeks)**	-	-	-	-	-	0.036
Up to 13	6/190	3.2	0.255	85	44.7	-
14–27	31/854	3.6	-	349	41.1	-
≥ 28	16/321	5.0	-	158	49.2	-
**Parity (*n* = 1495)**	-	-	-	-	-	0.001
Nil parity	14/461	3.0	0.004	142	30.8	-
1–2 parity	27/831	3.2	-	394	47.4	-
3–4 parity	15/186	8.1	-	114	61.3	-
≥ 5 parity	2/17	11.2	-	10	58.8	-
**HIV status (*n* = 1502)**	-	-	-	-	-	-
Negative	14/837	2.5	0.002	-	-	-
Positive	34/665	5.6	-	-	-	-
**On ART (*n* = 665)**	-	-	-	-	-	0.001
No	-	-	-	3	0.5	-
Yes	-	-	-	662	95.5	-
**RPR status at booking visit (*n* = 1499)**	-	-	-	-	-	0.002
Negative	-	-	-	62.8	43.5	-
Positive	-	-	-	37	63.8	-
**32 weeks RPR results (*n* = 1111)**	-	-	-	-	-	0.076
Negative	-	-	-	12	1.9	-
Positive	-	-	-	17	3.5	-

HIV, human immunodeficiency virus; RPR, Rapid Plasma Reagin; ART, anti-retroviral treatment.

Regression analysis (Tables 3) showed that teenage pregnant girls, 89% (OR = 0.11, 95% CI; 0.06:0.37, *p* < 0.05), ages 20–24 years, 81% (OR = 0.19, 95% CI; 0.11:0.33, *p* < 0.05) and ages 25–29 years, 62% (OR = 0.38, 95% CI; 0.23:0.65, *p* < 0.05) were less likely to be infected with HIV when compared to pregnant women of ≥ 35 years of ages. Pregnant women with GA between 14 and 27 weeks (second trimester) were 32% (OR = 0.68, 95% CI; 0.51:0.90, *p* < 0.05) less likely to be HIV positive as compared to those in the third trimester GA. Pregnant women who did not have syphilis were 55% (OR = 0.45, 95% CI; 0.22:0.92, *p* < 0.05) less likely to have HIV infection. Table 4 indicated that age was the only predictor for HIV incidence or seroconversion. Younger ages had lower odds ratios meaning that younger women were protective for HIV incidence. Age < 20 year, 88% (OR = 0.127, 95% CI; 0.02:0.63, *p* = 0.012) and age 20–24 years, 83% (OR = 0.17, 95% CI; 0.04:0.70, *p* = 0.015) were less likely to have HIV infection.

**TABLE 3:** Logistic regression output for human immunodeficiency virus positive at the booking visit.

Variables	*p*	Adjusted OR	95% CI for OR	
			Lower	Upper
**Age category (years)**	< 0.010	-	-	-
< 20	< 0.010	0.119	0.062	0.379
20–24	< 0.010	0.199	0.119	0.332
25–29	< 0.010	0.389	0.233	0.650
30–34	0.096	0.633	0.369	1.084
≥ 35	-	1.0	-	-
**Gestational age (weeks)**	0.024	-	-	-
Up to 13	0.364	0.835	0.566	1.232
14–27	0.007	0.682	0.515	0.902
≥ 28	-	1.0	-	-
**Parity**	0.009	-	-	-
Nil parity	0.816	0.875	0.285	2.693
1–2 parity	0.706	1.237	0.409	3.741
3–4 parity	0.360	1.704	0.544	5.331
≥ 5 parity	-	1.0	-	-
**Syphilis negative**	0.031	0.458	0.227	0.929
**Syphilis positive**	-	1.0	-	-
**Constant**	0.008	6.114	-	-
Reference group ≥ 35 years, Parity ≥ 5, GA ≥ 28 weeks and Syphilis positive. CI, confidence interval; OR, odds ratio.				

**TABLE 4:** Logistic regression output for incidence or seroconversion of human immunodeficiency virus infection.

Variables	*p*	Adjusted OR	95% CI for OR	
			Lower	Upper
**Age category (years)**	0.077	-	-	-
< 20	0.012	0.127	0.025	0.639
20–24	0.015	0.176	0.044	0.709
25–29	0.090	0.339	0.097	1.182
30–34	0.103	0.297	0.069	1.276
≥ 35	-	1.0	-	-

Reference group ≥ 35 years.

CI, confidence interval; OR, odds ratio.

### Seroprevalence, seroconversion, incidence and risk factors for syphilis

At the booking visit, 58 pregnant women had RPR reactive results thus estimating the prevalence of syphilis at 3.8% (95% CI; 3.1%:4.1%). All of them (58) received their first dose of penicillin treatment for being syphilis reactive. When we examined RPR results at the follow up visits (Table 1), we found RPR results of 1111 (74.1% of those who had negative results at booking visit) pregnant women and of them 29 (2.6%, 95% CI; 2.3%:2.8%) were RPR reactive (seroconversion). This resulted in follow up of 1111 pregnant women for 265 person-years with 29 new cases of syphilis with the incidence of syphilis of 10.9 (95% CI; 7.1:14.6) per 100 person-years. None of these new 29 RPR reactive cases were from HIV-positive women.

Ages of the pregnant women were significantly associated with positive syphilis status (*p* < 0.05) at the booking visit (Table 2). The prevalence of syphilis was lowest (1.5%) among the teenagers (age < 20 years) and the rates were found to increase with increasing ages of the pregnant women. Significantly, the highest rate (12.2%) of syphilis was among women ≥ 35 years of age. Gestational age was also significantly associated with syphilis status (*p* < 0.05). The highest prevalence of syphilis (5.0%) was found in pregnant women who were in the third trimester. Furthermore, parity was significantly associated with syphilis status. As the parity of the pregnant women increased, the prevalence of syphilis was also found to increase significantly (*p* < 0.05). Logistic regression output (Table 5) showed that age and HIV status were significant predictors for syphilis. Teenage (< 20 years) mothers 89% (OR = 0.11, 95% CI; 0.03:0.37, *p* < 0.05) and ages 20–24 years, 79% (OR = 0.21, 95% CI; 0.09:0.48, *p* < 0.05) less likely to have syphilis when compared with women ≥ 35 years of age. Pregnant women who were HIV negative were 56% less likely to have syphilis (OR = 0.44, 95% CI; 0.22:0.89, *p* < 0.05) than who were HIV positive. Table 6, logistic regression for incidence of syphilis showed that GA > 27 weeks were nine times (OR = 9.2, 95% CI; 1.17:72.40, *p* < 0.05) more likely to seroconvert or became newly infected with syphilis.

**TABLE 5:** Logistic regression output for syphilis at booking visit.

Variable	*p*	OR	95% CI for OR	
			Lower	Upper
**Age category (years)**	< 0.010	-	-	-
< 20	< 0.010	0.119	0.037	0.379
20–24	< 0.010	0.213	0.092	0.489
25–29	0.001	0.442	0.171	0.554
30–34	0.044	0.523	0.226	0.959
≥ 35	-	1.0	-	-
**HIV negative**	0.022	0.444	0.221	0.891
**HIV positive**	-	1.0	-	-
**Constant**	< 0.010	0.102	-	-

Reference group ≥ 35 years, HIV positive.

CI, confidence interval; OR, odds ratio.

**TABLE 6:** Logistic output for incidence or seroconversion of syphilis.

Variables	Significance (*p*)	Adjusted OR	95% CI for OR	
			Lower	Upper
**Gestational age coded (weeks)**	0.020	-	-	-
0–12	-	1	-	-
13–26	0.178	3.983	0.532	29.829
> 27	0.035	9.212	1.172	72.407
**Constant**	0.000	0.013	-	-

Reference group gestational age 0–12 weeks.

CI, confidence interval; OR, odds ratio.

## Discussion

The prevalence of maternal syphilis in our study is high (3.8%) at the booking visit. This is higher than the rates reported by the national surveys for SA (nationally 1.5% and 2%) and KZN (0.4% and 2.3%) during 2011 and 2015, respectively.^13,17^ The rate is also higher than the rate of 2.9% reported in a study of maternal syphilis in sub-Saharan Africa.^18^ Our study findings concur with the fact that there is a surge in the prevalence of maternal syphilis locally and globally.^19^ We find a higher rate of HIV infection (44.3%) in our population and HIV being a predictor for syphilis. It is found that pregnant women who are HIV positive had a significantly higher rate (5.6%) of syphilis compared to 2.5% for pregnant women who are HIV-negative. The result was an OR = 0.44 for pregnant women who are HIV-negative. However, the seroconversion rate of maternal syphilis is 2.6% in contrast with the rate reported from Tanzania (2.7%).^20^ Both higher rates of syphilis at booking visit and seroconversion of our study can be explained partly with the higher prevalence of HIV in our setting.^12^ Similarly, the incidence of syphilis in our study of 10.9 per 100 person years is high. However, when comparing with the recent report from SA (2021) on the incidence of any sexually transmitted infection (STI) during pregnancy and the early post-partum period, our study finding is lower than 15 infections per 100 women-years.^21^

Similarly, the higher incidence of STI is estimated at 20 per 100 women years from Durban.^22^ This higher rate of syphilis prevalence, seroconversion and incidence thus could largely be related to a higher rate of HIV in our population. Maternal syphilis is a known pivotal cause of perinatal morbidity and mortality. However, it is not clear whether the high rate of syphilis could be as a result of risky sexual behaviour of pregnant women. Therefore, regular testing and treatment for syphilis are critical during ANC and further research is needed to identify those risky behaviours.

The result of syphilis seroconversion and incidence rates should be interpreted with caution as not every pregnant woman who attended KCHC for their first antenatal visit and who was syphilis negative had a retest done at or around 32 weeks of gestation. The follow up rate was 77%. It was possible that healthcare workers might overlook or forget to conduct a rescreening test for syphilis and or recording in the ACR. This may be a reason why some women are missing syphilis rescreening results. A possible explanation could be that the pregnant women could have experienced an array of complications during the antenatal period and would thus have been referred to a higher care institute for antenatal and delivery care. It might also be possible that some pregnant women who attended KCHC on their booking ANC visits either changed to different healthcare facilities, experienced loss of pregnancy or delivered prematurely. The actual rates of pregnancy complications and reasons for referrals to hospital and lost to follow-up during the antenatal period were not assessed in this study. However, it is known that approximately 60% – 70% of all pregnant women who utilise a PHC facility in public health settings in SA would most likely require the service of a hospital at some point in their antenatal period.^16^ However, HIV retest rate after 12 weeks (3 months) was good of 95%.

The present study found a significant association between the age of the pregnant women and both HIV and syphilis infections among these pregnant women. Older pregnant women had a higher rate of these infections compared to younger ones. Reports from other parts of Africa had shown that the prevalence of syphilis was found to increase from 0.6% in the younger age group to 1.1% in the older women in Rwanda, 0.9% in the younger women which increased to 3.7% in the older women in Uganda and in Kenya where the rate was 0.9% in the younger as compared to 2.5% in the older pregnant women.^22,23,24^ We are uncertain as to why maternal syphilis is more prevalent in the older age groups, as sexually transmitted infections are known to occur commonly in younger women and this finding warrants further investigation.^14^

Similar to syphilis, the prevalence of HIV among younger pregnant women (age ≤ 29 years) are found to decline from 21.8% in 2010 to 18.5% in 2017 in SA.^12^ Thus our findings concurred with the latest SA report on age distribution of HIV infection among pregnant women from the annual national antenatal HIV seroprevalence survey (2017) which showed that the prevalence of HIV is at a lower rate among younger women (< 29 years) than the older women.^12^ This trend of decreasing HIV prevalence among younger pregnant women of < 24 years of ages and increasing among the older age > 35 years group was also found from another report from Africa.^25^

This co-infection rate is considered lower than the rate of 10% reported from the multi-country study conducted in SA, Brazil, Argentina and the USA.^26^ Low socio-economic status of pregnant mothers, the GA (advanced GA) when syphilis was not treated, and having multiple sexual partners were all found to contribute to deliver an infant infected with syphilis from a HIV-infected mother.^26^ The co-infection of syphilis and HIV was not well understood with limited research to describe the actual burden of syphilis in women living in poor socioeconomic conditions. However, it was known that infection with syphilis increases the viral load among HIV infected individuals and also decreases the CD4+ T lymphocytes.^18^

We estimated a higher prevalence of HIV (44.3%) among our study population. This is higher than the rate estimated for KZN (41.1%) in 2017 during the annual survey.^12^ But this rate was similar to the earlier findings (2015) of 44.4% for KZN and a later report from Cape Town.^17,27^ The seroconversion rate of HIV (4%) at 12 weeks interval of the booking visit is higher than a rate of 2% found in Tanzania.^20^ The higher rate of HIV seroconversion can be justified with a higher seroprevalence of HIV (44.3%) in our study compared to a much lower prevalence of 7.2% in Tanzania.^20^ A higher seroconversion rate (9.2%) was found between the booking visit and the time of delivery when compared to an earlier study conducted in Durban SA.^28^ Lower HIV seroconversion rates were found as 1.2% and 1.5% at 3 months follow up and at the time of delivery from the booking visit, respectively, at a regional hospital in Durban, SA.^19^ The seroconversion rate of HIV (4%) at 12 weeks interval of the booking visit is higher than a rate of 2% found in Tanzania.^20^ The incidence of HIV infection of 17.3 per 100 person-years in our study is much higher than the incidence estimated from a meta-analysis of 19 cohort studies of pregnant women of 4.7 per 100 person-years.^29^ In the general population of rural KZN, it is reported in 2017 that the female HIV incidence was 3.06 (2.38–3.94) seroconversion events per 100 person-years that is higher than male incidence.^30^ A much lower annual HIV incidence rate of 1.5 cases per 100 person-years is also reported in 2017.^31^ The high incidence of both HIV and syphilis in our study could be because of short time follow up.

We found that pregnant women who are syphilis negative, are 55% (OR = 0.459, *p* < 0.05) less likely to have HIV infection. This is similar to the findings of another report from Durban.^19^

Therefore, it is seen that the prevalence of syphilis infection can increase HIV infection in pregnant women. It is already known that the syphilis infection can increase HIV transmission to the foetus two to four-fold, as in mothers infected with syphilis is significantly associated with HIV transmission from mother to the unborn child.^26^ Thus, it is imperative that all pregnant women, more so those women co-infected with HIV are repeatedly screened for syphilis if co-infection is present. These women should also receive treatment appropriately and timeously to decrease the risk of vertical transmission of HIV and syphilis to the newborn infant.

Analysis of risk factors for HIV had shown that older age of pregnant women, advance in pregnancy (last trimester), higher parity and lastly the infection with syphilis are associated with higher rate of HIV in our study population. These higher rates of syphilis and HIV infection among pregnant women suggest that these infections continue to exist as an important public health concern with the hypothesis that these prevalence rates do vary at different geographical areas and may have masked the national and provincial rates. Thus, there is a need to strengthen intervention efforts at the institutional and or community levels.

This study has some limitations namely, the retrospective nature and limited study variables for risk factors, errors in data abstraction, selection bias because of loss to follow up. We only studied those pregnant women who attended our ANC clinic. Our rates might have underestimated or overestimated the prevalence of syphilis and HIV as a significant number of women were missing because of inaccessibility to the ANC clinic or as a result of personal, behavioural or socio-economic (cost to travel) reasons. However, it is known that a high proportion (> 95%) of pregnant women do attend ANC at least once in KZN.^32^

## Conclusion

Our study finds higher prevalence, incidence and seroconversion rates of HIV and syphilis among pregnant women. Maternal age, parity, and HIV status were found to be risk factors for syphilis. Similarly, maternal age, GA, parity, and syphilis were risk factors for maternal HIV. Higher rates of seroconversion and incidence of HIV and syphilis suggest that these women may potentially transmit these infections to the unborn babies and affect the pregnancy outcomes negatively. The strategies should be strengthened to local areas and health facilities including counselling and testing, ART and health education to change the behaviour on prevention of HIV and syphilis among pregnant women in particular and the general population at large. Further studies are recommended to identify the behavioural factors of pregnant women related to higher rates of HIV and syphilis infections among high-risk groups.
